# Supplementary material for: A variably imprinted epiallele impacts seed development
Source: PLoS Genet. 2018 Nov 5;14(11):e1007469. doi: 10.1371/journal.pgen.1007469 (PMC6237401; doi:10.1371/journal.pgen.1007469)
Supplement: S1 Fig — (PDF) [file pgen.1007469.s001.pdf]

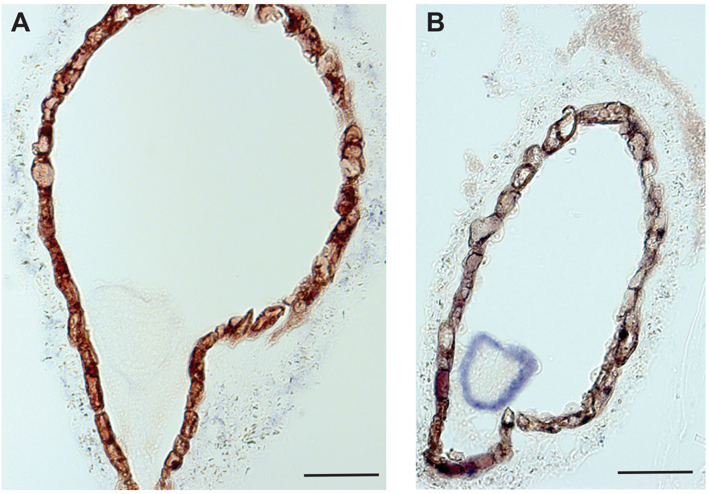

S1 Fig. Negative and positive controls for *in situ* hybridization. (A) *HDG3* sense probe hybridization for Col x Col seed. (B) *PDF1* antisense probe hybridization for *hdg3-1* x *hdg3-1* seed. Scale bars, 50 μm.
