# Supplementary material for: A variably imprinted epiallele impacts seed development
Source: PLoS Genet. 2018 Nov 5;14(11):e1007469. doi: 10.1371/journal.pgen.1007469 (PMC6237401; doi:10.1371/journal.pgen.1007469)
Supplement: S2 Fig — (PDF) [file pgen.1007469.s002.pdf]

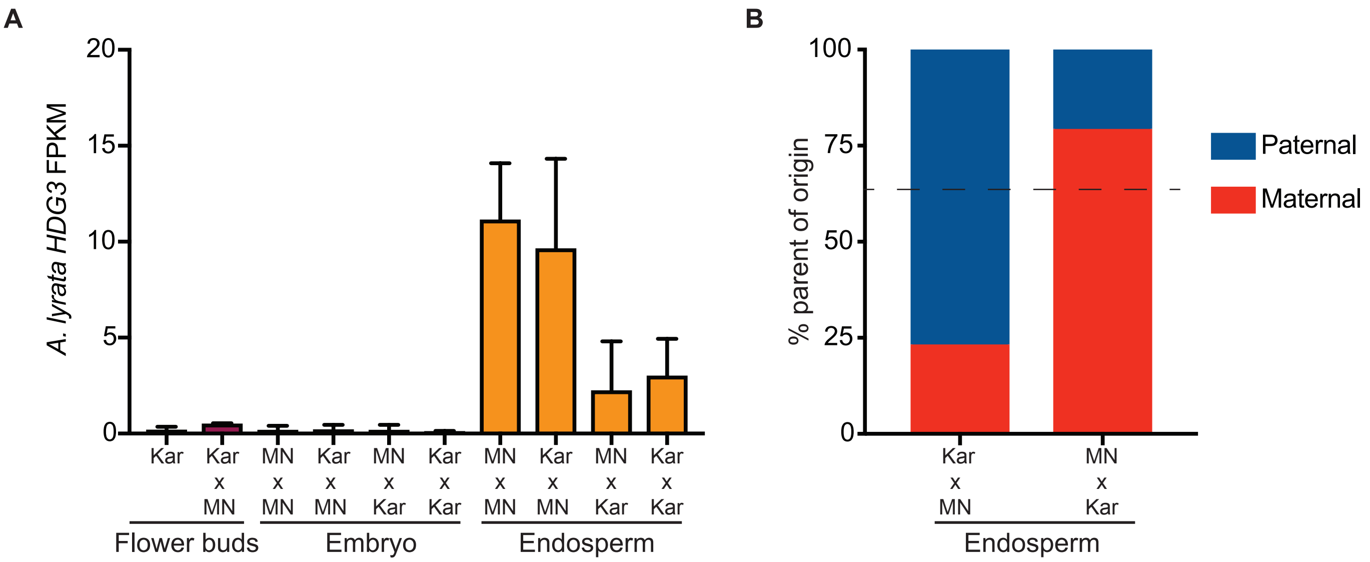

S2 Fig. *HDG3* expression in *A. lyrata*. (A) Expression of *HDG3* (ALAl_scaffold_0004_1698 on scaffold 4:15306009-15309421) is specific to endosperm. Bars show mean FPKM values with std deviation for 2-4 biological replicates per genotype. (B) Percent maternal and paternal allele transcripts for the indicated crosses. All data are culled from [8].
