# Supplementary material for: A variably imprinted epiallele impacts seed development
Source: PLoS Genet. 2018 Nov 5;14(11):e1007469. doi: 10.1371/journal.pgen.1007469 (PMC6237401; doi:10.1371/journal.pgen.1007469)
Supplement: S3 Fig — (PDF) [file pgen.1007469.s003.pdf]

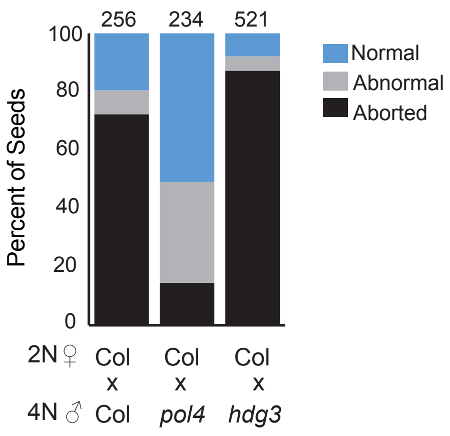

S3 Fig. Tetraploid *hdg3-1* does not rescue interploidy seed lethality caused by paternal genomic excess. Tetraploid plants were created by colchine treatment and confirmed by flow cytometry analysis of DNA content. Tetraploid *pol iv* mutants are a positive control for interploidy seed rescue. Number of seeds analyzed on top of bars.
