# Supplementary material for: A variably imprinted epiallele impacts seed development
Source: PLoS Genet. 2018 Nov 5;14(11):e1007469. doi: 10.1371/journal.pgen.1007469 (PMC6237401; doi:10.1371/journal.pgen.1007469)
Supplement: S4 Fig — (PDF) [file pgen.1007469.s004.pdf]

A

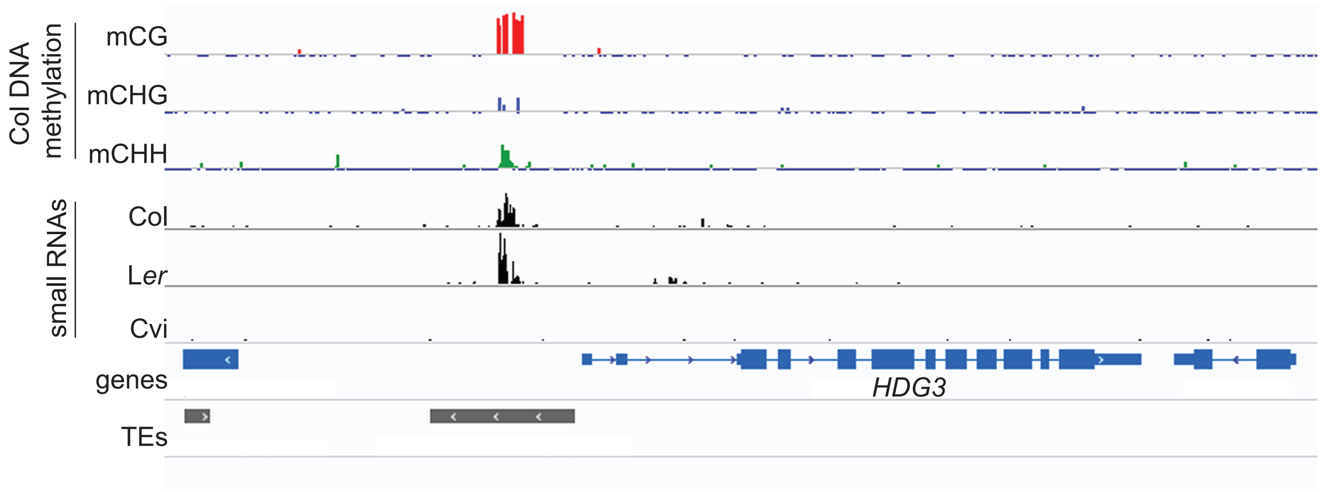

B

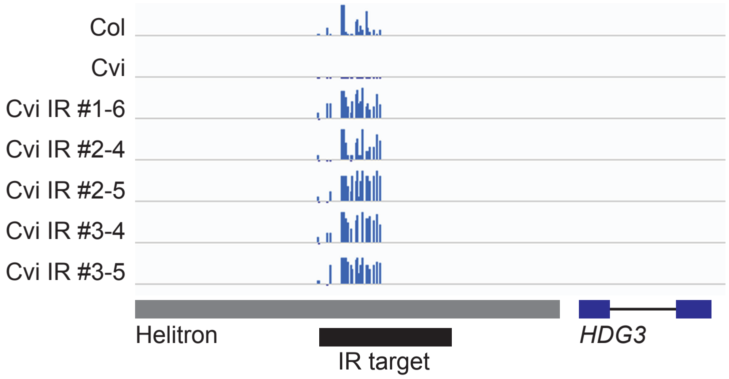

C

Tissue	# clones	% CG	% CHG	% CHH
Col leaf	15	80	24	14
Cvi leaf	11	1	0	0
Cvi IR #1-6 leaf	10	94	73	38
Cvi IR #2-4 leaf	7	96	38	21
Cvi IR #2-5 leaf	6	93	72	48
Cvi IR #3-4 leaf	15	99	69	51
Cvi IR #3-5 leaf	8	90	75	46

S4 Fig. Gain of *HDG3* methylation in Cvi *HDG3* IR lines. (A) Cvi naturally lacks small RNAs at the TE 5' of *HDG3*. DNA methylation data is from Col embryos (data from [20]); small RNA data is from Col, L*er* and Cvi embryos (data from [31]). (B) Total DNA methylation in Col, Cvi, and Cvi *HDG3* IR leaves as determined by bisulfite-PCR. Scale is 100%, tick marks below line indicate unmethylated cytosines. (C) Quantification of above data.
