# Supplementary material for: A variably imprinted epiallele impacts seed development
Source: PLoS Genet. 2018 Nov 5;14(11):e1007469. doi: 10.1371/journal.pgen.1007469 (PMC6237401; doi:10.1371/journal.pgen.1007469)
Supplement: S5 Fig — (PDF) [file pgen.1007469.s005.pdf]

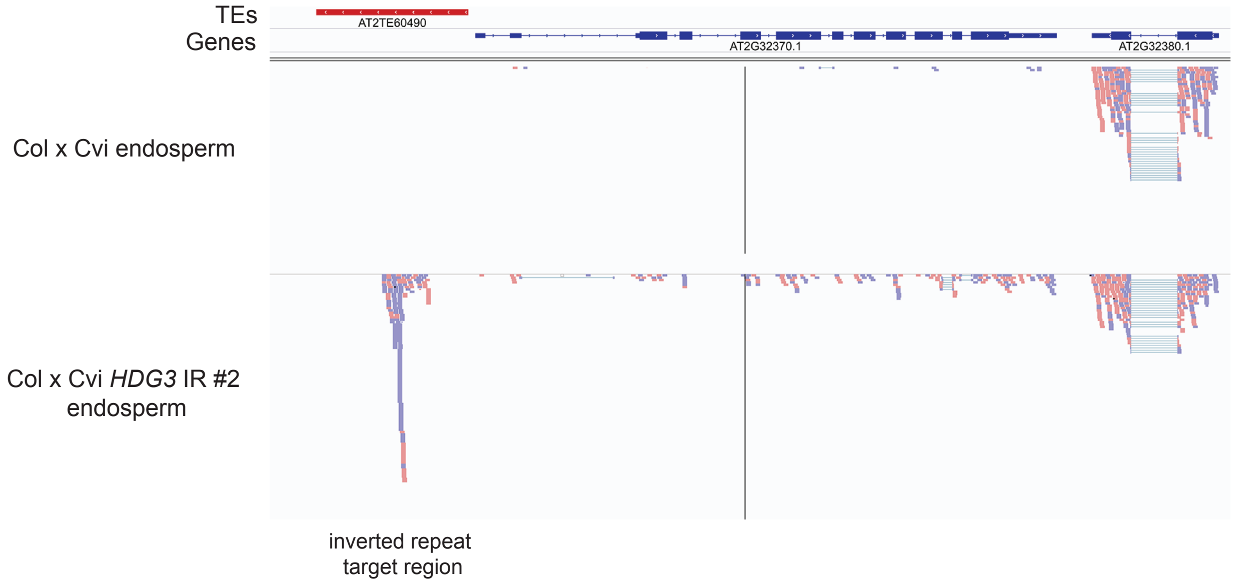

S5 Fig. Accumulation of inverted repeat RNA in Col x Cvi *HDG3* IR endosperm. Mapping of mRNA-seq reads to the *HDG3* (AT2G32370) locus in Col x Cvi and Col x Cvi *HDG3* IR #2 endosperm. Reads that match the inverted repeat target region represent expression of the inverted repeat transgene in endosperm from a different location.
