# Supplementary material for: A variably imprinted epiallele impacts seed development
Source: PLoS Genet. 2018 Nov 5;14(11):e1007469. doi: 10.1371/journal.pgen.1007469 (PMC6237401; doi:10.1371/journal.pgen.1007469)
Supplement: S6 Fig — (PDF) [file pgen.1007469.s006.pdf]

A

Cross	Tissue	Allele	# Clones	% CG	% CHG	% CHH
Cvi x Col	Endosperm	Col	14	77.6	9.5	4.8
		Cvi	9	1.6	0	0
	Embryo	Col	9	74.1	3.7	7
		Cvi	18	2.3	0	2.5
Col x Cvi	Endosperm	Col	14	34.5	9.5	0.4
		Cvi	9	0	0	0.6
	Embryo	Col	16	87.5	14.6	3.6
		Cvi	6	2.4	5.6	0
Col x Cvi IR #1	Endosperm	Col	20	16	13.6	9.5
		Cvi	9	64.5	66.7	35.9
Col x Cvi IR #2	Endosperm	Col	31	31	14.0	5.7
		Cvi	21	18	19.1	28.2
	Embryo	Col	11	100	42.4	35.9
		Cvi	17	92.2	37.3	32
Col x Cvi IR #3	Endosperm	Col	8	30	24.0	17.4
		Cvi	23	86.1	69.6	40.6

B

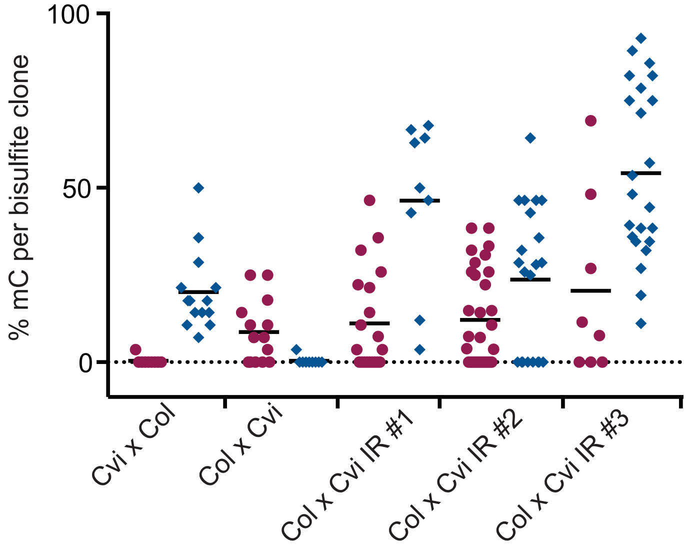

S6 Fig. Bisulfite-PCR analysis of *HDG3* methylation in seeds. (A) % methylation from the indicated crosses. Female parent listed first. IR lines are independent transgenic events. Cvi x Col and Col x Cvi data are from [20]. (B) Endosperm total DNA methylation (%) for each bisulfite clone from the above data. Maroon circles, maternal allele clones; blue diamonds, paternal allele clones.
