# Supplementary material for: A variably imprinted epiallele impacts seed development
Source: PLoS Genet. 2018 Nov 5;14(11):e1007469. doi: 10.1371/journal.pgen.1007469 (PMC6237401; doi:10.1371/journal.pgen.1007469)
Supplement: S2 Table — (PDF) [file pgen.1007469.s008.pdf]

S2 Table: Endosperm Cellularization Timing and Embryogenesis Stages at 5 DAP

Cellularization: Delayed ← | Normal | Early →

Col x Col	Delayed			Normal			Early					Stage Total
Torpedo												0
Late Heart												0
Heart			2									2
Early Heart				15								15
Triangle				13								13
Late Globular												0
Globular				1								1
Early Globular												0
Pre Globular (4 cell)												0
Timing Totals	0	0	2	29	0	0	0	0	0	0	0	31

Col x Cvi	Delayed			Normal			Early					Stage Total
Torpedo												0
Late Heart					1	1						2
Heart				22	5	3	9					39
Early Heart				2	2							4
Triangle												0
Late Globular				1								1
Globular				3		1				1		5
Early Globular				1						1		2
Pre Globular (4 cell)												0
Timing Totals	0	0	0	29	8	5	9	0	0	2	0	53

Cvi x Cvi	Delayed			Normal			Early					Stage Total
Torpedo												0
Late Heart												0
Heart												0
Early Heart												0
Triangle				10								10
Late Globular				23								23
Globular				222								222
Early Globular				17								17
Pre Globular (4 cell)												0
Timing Totals	0	0	0	272	0	0	0	0	0	0	0	272

hdg3-1 x *hdg3-1*	Delayed			Normal			Early					Stage Total
Torpedo												0
Late Heart												0
Heart		4	5									9
Early Heart			24	1								25
Triangle			6	5								11
Late Globular				2	1							3
Globular												0
Early Globular							1					1
Pre Globular (4 cell)												0
Timing Totals	0	4	35	8	1	0	1	0	0	0	0	49

Col x Cvi IR 2-5	Delayed			Normal			Early					Stage Total
Torpedo												0
Late Heart												0
Heart							3	2		2	1	8
Early Heart							2	2	2	2	3	11
Triangle						2	2	2	2	2	1	11
Late Globular						1				1	1	3
Globular										5	2	7
Early Globular								1	1		1	3
Pre Globular (4 cell)												0
Timing Totals	0	0	0	0	0	3	7	7	5	12	9	43

Cvi x Cvi IR 2-5	Delayed			Normal			Early					Stage Total
Torpedo												0
Late Heart						1						1
Heart					2							2
Early Heart				3	3							6
Triangle				4	4	2						10
Late Globular				3	1							4
Globular				2	1							3
Early Globular				2								2
Pre Globular (4 cell)												0
Timing Totals	0	0	0	14	11	3	0	0	0	0	0	28

Col x *hdg3-1*	Delayed			Normal			Early					Stage Total
Torpedo												0
Late Heart												0
Heart			3									3
Early Heart			9	1								10
Triangle			8	2								10
Late Globular				1								1
Globular				1								1
Early Globular												0
Pre Globular (4 cell)												0
Timing Totals	0	0	20	5	0	0	0	0	0	0	0	25

Col x Cvi IR 3-4	Delayed			Normal			Early					Stage Total
Torpedo												0
Late Heart					1				1	1		3
Heart				4		3	1	3	1	2	1	15
Early Heart				1					1	2	1	5
Triangle				1	1				3	1		6
Late Globular										1		1
Globular								1	2	4	1	8
Early Globular											1	1
Pre Globular (4 cell)											1	1
Timing Totals	0	0	0	6	2	3	1	4	8	11	5	40

Cvi x Cvi IR 3-4	Delayed			Normal			Early					Stage Total
Torpedo												0
Late Heart												0
Heart												0
Early Heart						5						5
Triangle					3	1						4
Late Globular					2	1						3
Globular												0
Early Globular												0
Pre Globular (4 cell)												0
Timing Totals	0	0	0	0	5	7	0	0	0	0	0	12

hdg3-1 x Col	Delayed			Normal			Early					Stage Total
Torpedo												0
Late Heart				1								1
Heart			1	10	3							14
Early Heart				21	3							24
Triangle				25	3	1						29
Late Globular				8	1							9
Globular												0
Early Globular												0
Pre Globular (4 cell)												0
Timing Totals	0	0	1	65	10	1	0	0	0	0	0	77

hdg3-1 x Cvi IR 2-5	Delayed			Normal			Early					Stage Total
Torpedo											1	1
Late Heart												0
Heart							1	1		3		5
Early Heart									2	3	1	6
Triangle						3		1	1		1	6
Late Globular											3	3
Globular						1				3	6	10
Early Globular												0
Pre Globular (4 cell)												0
Timing Totals	0	0	0	0	0	4	1	2	3	9	12	31

Cvi x Col	Delayed			Normal			Early					Stage Total
Torpedo												0
Late Heart												0
Heart												0
Early Heart												0
Triangle												0
Late Globular				5								5
Globular				54								54
Early Globular				24								24
Pre Globular (4 cell)				5								5
Timing Totals	0	0	0	88	0	0	0	0	0	0	0	88
