# Supplementary material for: A variably imprinted epiallele impacts seed development
Source: PLoS Genet. 2018 Nov 5;14(11):e1007469. doi: 10.1371/journal.pgen.1007469 (PMC6237401; doi:10.1371/journal.pgen.1007469)
Supplement: S3 Table — (PDF) [file pgen.1007469.s009.pdf]

S3 Table. Tests of statistical significance for pairwise comparisons of differences in endosperm or embryo development.

Seed Genotype 1	Seed Genotype 2	Endosperm diff?	Embryo diff?
Col x Col	*hdg3* x *hdg3*	***	
Col x Col	Col x *hdg3*	***	
Col x Col	*hdg3* x Col		
hdg3 x Col	Col x *hdg3*	***	
Col x Cvi	Col x Cvi *HDG3* IR 2-5	***	***
Col x Cvi	Col x Cvi *HDG3* IR 3-4	***	*
Col x Cvi	*hdg3* x Cvi *HDG3* IR 2-5	***	***
Cvi x Cvi	Cvi x Cvi *HDG3* IR 2-5		***
Cvi x Cvi	Cvi x Cvi *HDG3* IR 3-4		***
Col x Col	Col x Cvi	**	***
Col x Cvi	Cvi x Col	***	***
Col x Col	Cvi x Cvi	***	***
Col x Cvi	Cvi x Cvi	***	***
Cvi x Col	Cvi x Cvi		
Col x Col	Cvi x Col	*	***

***= difference significant, padj < 0.001; **= difference significant, padj < 0.01; *=difference significant, padj < 0.05; no stars = no significant difference
