# Supplementary material for: A variably imprinted epiallele impacts seed development
Source: PLoS Genet. 2018 Nov 5;14(11):e1007469. doi: 10.1371/journal.pgen.1007469 (PMC6237401; doi:10.1371/journal.pgen.1007469)
Supplement: S5 Table — (PDF) [file pgen.1007469.s011.pdf]

S5 Table. Oligos used in this study.

Oligo Name	Sequence 5'-3'	Gene	Purpose
Expression			
HDG3 Fwd Set 4	CATCATGGAGTTGGCATTTGG	*HDG3*	TaqMan RT-qPCR
HDG3 Rev Set 4	CAAAGCTAAGCTAGTGCCATTAAA	*HDG3*	TaqMan RT-qPCR
HDG3 Prb Set 4	/56-JOEN/ACT TGA GCC/ZEN/ ATC ACC AAG AGC TCC/3IABKFQ/	*HDG3* – Col	Col probe for TaqMan RT-qPCR
HDG3_Cvi	/56-FAM/ACT TGA GTC /ZEN/ ATC ACC AAG AAC TCC/3IABkFQ/	*HDG3* – Cvi	Cvi probe for Taqman RT-qPCR
MG510	TGCAACGGAGAGATGATGCACAAG	*HDG3*	Sybr Green RT-qPCR
MG511	TGCTCTTGCTAGTGTGTCCATGCC	*HDG3*	
MG446	CCATTCTACTTTTTGGCGGCT	AT1G58050	Sybr Green RT-qPCR
MG447	TCAATGGTAACTGATCCACTCTGATG	AT1G58050	
DNA methylation			
MG422	GTTTAAGGATATTTTGGATAATGTATTGA	*HDG3* 5' TE	BS-PCR
MG423	CTATRCTTTTATTAACTATATARATCRTTATACAC	*HDG3* 5' TE	
MG424	TACATCTCATATCTACAAATARTATTATTAAC	*HDG3* 5' TE	BS-PCR
MG425	TGGTATGAGYYTAGGAGAAATAATGTAAG	*HDG3* 5' TE	
In situ			
KN13	TCTCACCCTTCACCTCCATC	*PDF1*	Amplify 602 bp of *PDF1* coding sequence for in situ probe
KN14	GGGGTTGTGAAAGGGAACTT	*PDF1*	
KN19	GATGGGATCTAAGGGAAATGTCG	*HDG3*	Amplify 278 bp of *HDG3* coding sequence for in situ probe
KN20	ACATTGCCACGAGTGCAGTT	*HDG3*	
IR cloning			
DP215	CACCTACCAAATTATTATCTATTGAT	*HDG3* 5' TE	Amplify IR sequence for cloning
DP216	GAATTTCATAGTGAAATGGACCATC	*HDG3* 5' TE	
